# Evaluating the effect of an adapted mental health literacy intervention on mental health related stigma among secondary students in Germany: results of a pre-post evaluation study

**DOI:** 10.1186/s12889-023-16825-y

**Published:** 2023-10-10

**Authors:** Sandra Kirchhoff, Alexandra M. Fretian, Orkan Okan, Ullrich Bauer

**Affiliations:** 1 Department of Sport and Health Sciences, Technical University of Munich, 80992 Munich, Germany; 2 Faculty of Educational Science, Bielefeld University, 33615 Bielefeld, Germany; 3 Centre for Prevention and Intervention in Childhood and Adolescence (CPI), Faculty of Educational Science, Bielefeld University, 33615 Bielefeld, Germany

**Keywords:** Stigma, Mental health literacy, Intervention, Adolescents, Mental health, School

## Abstract

**Background:**

Most mental health problems develop during youth, with about three quarter emerging before age 25. In adolescence, stigmatizing attitudes related to mental illness become more nuanced and consolidate into one’s belief system. As the stigma of mental illness is still one of the leading barriers to help-seeking, intervention measures should explicitly address it before it becomes entrenched over time. Preventive measures, for example, based on promoting mental health literacy (MHL), can be used to address and tackle stigmatizing attitudes. The Canadian MHL-based intervention “the Guide” was translated and adapted for the use in German schools. The present study evaluates the effect of the German version of *the Guide* on attitudes towards mental illness among students in Germany.

**Methods:**

The first-time application of *the Guide* (German version) was evaluated with a pre-post-evaluation study with an intervention and a control group. The evaluation data of 188 students (intervention group n = 106, control group n = 82) were statistically analyzed focusing on the outcomes social stigma, social distance, and self-stigma.

**Results:**

The analysis showed that participants do not tend to hold stigmatizing attitudes even before the intervention. Nevertheless, the intervention was effective in reducing social stigma, but not in reducing social distance and self-stigma. Neither gender, pre-existing experience with mental illness, nor the delivery modality of the contact element within the intervention (speaker vs. video) seemed to influence the outcomes.

**Conclusions:**

The German version of the MHL-based intervention, *the Guide*, seems to be a suitable intervention to improve attitudes towards mental illness among students in Germany. More extensive research is necessary to confirm the findings and further explore factors that influence the program’s effects on attitudes short- and long-term.

**Supplementary Information:**

The online version contains supplementary material available at 10.1186/s12889-023-16825-y.

## Background

Youth is a developmental period in which the first diagnosis of mental disorders becomes prominent, with 50% of all mental illnesses emerging before age 14 and up to 70–75% by age 25 [1]. Untreated and delayed treatment of mental illness can exacerbate symptomatology itself (especially for schizophrenia and affective disorders) [2, 3] and worsen relevant social determinants of mental illness (such as school dropout [4], economic insecurity, etc.) and thus uphold a negative feedback loop, in which multiple disadvantages accumulate over time [5]. Various factors that prevent young people from mental health help-seeking have been identified, among the most relevant being: limited mental health knowledge, stigma, and structural issues related to the accessibility of mental health services [6]. Different approaches were created to tackle the barriers of mental health help-seeking. Generally, they address varying, interrelated dimensions, such as mental health knowledge, stereotypes, help-seeking attitudes, intentions, or behaviors (see systematic reviews from Salerno et al. 2016 [7] and Wei et al. 2013 [8]), alongside different forms of stigma. These approaches have different foci and can be found under different labels, such as “anti-stigma”-, “mental health awareness”-, or “mental health literacy”-interventions. The effectiveness of these interventions varies slightly based on the measured outcomes: small to moderate positive changes have been found in mental health knowledge [9, 10], while small changes were registered for stigma and help-seeking [7, 9]. Although this trend was confirmed by multiple reviews [7, 9], a recently published review provided conflicting results with regard to stigma and help-seeking, which did not change significantly [11].

This speaks for the need of evidence-based interventions that successfully improve the mentioned dimensions. One program that has proven to be internationally successful is the Canadian school-based intervention called “the Guide”. Various evaluation studies report the effectiveness of this mental health literacy-based intervention, especially in increasing mental health knowledge and decreasing stigmatizing attitudes among adolescents. This has proven to be consistent across countries and different school types (e.g., [12–16]). The term mental health literacy (MHL) encompasses capacities and resources that enable and strengthen people in taking care of their mental health and address documented barriers to help-seeking (e.g., stigmatizing attitudes, lack of mental health knowledge and help-seeking strategies [6, 17]). Four components are part of MHL: (1) understanding how to obtain and maintain positive mental health; (2) understanding mental disorders and their treatments; (3) decreasing stigma related to mental disorders; and (4) enhancing help-seeking efficacy [18].

While a few school prevention programs addressing mental health exist in Germany (e.g., “Mind Matters” [19], “Crazy? So what!” [20]) none of them focus on strengthening mental health literacy. To complement the existing landscape of interventions in Germany, the *the Guide* intervention was translated and adapted for the German school context, and evaluated to verify its effectiveness in improving different dimensions of MHL. While the analysis of the intervention’s effects on mental health knowledge and help-seeking efficacy was recently published elsewhere [21], this study focuses on its effects on stigma.

Addressing stigma early in the life course is necessary. Research shows that stigmatizing beliefs gradually emerge and be endorsed around middle childhood, for example, by associating more negative labels to people with mental illness than other groups (e.g., those with physical illness). With the transition into adolescence, stigmatizing views become more sophisticated and nuanced [22]. Thus, tackling and molding stigmatizing attitudes before they become a rigid belief system has been argued to be more effective in this period [22]. Stigma is a multifaceted socially constructed concept that can be measured and categorized in various ways. Research differentiates between structural stigma (stigma perpetrated through social institutions/systems), public or social stigma (misconceptions/prejudices of the public that can lead to stigmatizing action), anticipated stigma (stigma felt when stigmatized person fears negative reactions), associative stigma (stigma experienced by associates of stigmatized persons), self-stigma (internalization/application to self of public negative beliefs), and others [23, 24].

Individual-focused preventive measures, such as the MHL-based intervention *the Guide*, primarily aim at reducing stigma on a personal level. Accordingly, this study focusses on the following three different stigma types that are expected to be improved by the MHL-intervention. To assess public/social stigma, misconceptions and prejudices of young people around mental illness were measured. Additionally, a more behavioral-oriented measure was applied, which expresses the desire to exclude people with mental illness from various social situations. This is called social distance, and lower levels have been linked to greater reported contact with people with mental illness, thus indicating less stigma [25]. Further, to verify if negative beliefs have been internalized, a measure of self-stigma was included.

Thus, the objective of this study is to examine whether the adapted MHL-based intervention (*the Guide* – German version) generates improvements in attitudes towards mental illness (particularly social stigma, social distance, and self-stigma) among adolescents in Germany. The secondary goals are to explore whether the expected changes in the intervention group are related to relevant demographic or other characteristics known to be associated with stigma from past research, such as social desirability or knowing someone with mental illness.

## Methods

### Mental health literacy intervention “the Guide” (German version)

The German version of *the Guide* (German title: “Psychische Gesundheit und Schule – Unterrichtsprogramm”) [26] is the official German adaptation of the “Mental Health and High School Curriculum Guide (*the Guide*)” intervention [27]. The translation to German language and adaption to the German school setting was realized as part of the German research project “IMPRES”, which is a subproject of the “Health Literacy in Childhood and Adolescence (HLCA)” research consortium funded by the Federal Ministry of Education and Research and conducted by Bielefeld University (03/2018-04/2022) and the Technical University of Munich (04/2022-12/2022). The objective of the IMPRES project was to develop, implement and evaluate a mental health literacy intervention targeting youth to foster mental health capacities and reduce stigmatization effectively. The project was actively supported by local cooperation partners from the school administration and school counselling field, the mental health care coordination, an association of people with lived experience with mental illness, and a non-profit foundation.

*The Guide* intervention is a curriculum program with comprehensive teaching materials on mental health and mental illness created by Canadian researchers and experts on adolescent mental health, Dr. Stanley Kutcher and Dr. Yifeng Wei. It can be used by classroom teachers to educate youth and strengthen personal capacities in terms of mental health literacy (MHL) [18]. The main target group of *the Guide* is students aged 13 to 15 [27], which translates to the target group of 8th to 10th graders in Germany. The original program handbook, the adapted German version, and adaptations to other languages, e.g., French, Spanish, or Chinese, are freely available online^1^.

Just like the original, the German version [26] comprises six modules that address MHL’s abovementioned components. The modules deal with (1) stigma of mental illness, (2) understanding mental health and mental illness, (3) information on specific mental illnesses, (4) experiences of mental illness, (5) seeking help and finding support, and (6) the importance of positive mental health. In particular, modules 1 and 4 address attitudes and beliefs about mental health and aim at reducing stigmatization. Reports of personal experiences with mental illness are being discussed to foster students’ empathy and acceptance and tackle existing reservations and prejudices. The 4th module, which focuses on lived experiences with mental illness, entails two alternatives to deliver the contact element. Video-based digital stories from young people can be used (indirect contact) or speakers can be invited to class to talk to the students about their experiences with mental illness (direct contact).

The contents and activities of each module are described in detailed lesson plans to facilitate the delivery by school teachers. The program comprises different teaching materials and methods (working sheets, videos, PowerPoint presentations, activity cards, group activities, discussions, etc.) and enables interactive learning on mental health. Apart from core contents and activities, additional materials are available. The delivery of the core contents requires a minimum of 7–8 school hours.

### Procedure

The first-time application and pilot testing of the German version of *the Guide* started in the fall of 2019 in the region of Bielefeld, Germany. Local secondary general and vocational schools were informed about the mental health teaching program via e-mail and telephone and were invited to participate. To facilitate the program implementation, preparatory one-day trainings on teaching *the Guide* (German version) were offered. The training was realized four times by the project team between October 2019 and April 2021. In total, 32 teachers and social workers from general and vocational schools participated in the trainings. Participants were provided with a physical material folder containing the printed teaching materials (handbook describing the use of the materials, background information about mental health and mental illnesses, detailed teaching instructions of the modules, and additional materials) and a UBS flash drive comprising digital materials (e.g., presentation slides, videos).

Both the participation in the training and the implementation of the program at the respective school were voluntary. Teachers interested in implementing the program were given the opportunity to invite a speaker to class as part of module 4. The preparatory work was organized by the project team and collaborating partners. Most speakers who took part in the delivery of the intervention were actively involved with the cooperating association of people with lived experience with mental health. They were contacted through the association and invited to participate in the program. Prior training and ongoing support throughout their participation were offered to ensure speakers were prepared and felt safe visiting a class and sharing their personal stories.

Participating teachers were instructed to maintain fidelity to the core contents of the modules and complete the mandatory module activities. They were otherwise given the flexibility to include any additional materials marked as “optional” in the program. In addition, they were asked to briefly document the classroom delivery of the program indicating which module activities they realized and which they did not.

### Study design

To evaluate the effects of the German version of *the Guide* on stigmatizing attitudes at the student level, an evaluation study was conducted using a pre-post intervention and control group design. Groups were formed by convenience. Participating students of the intervention group (IG) were asked to complete a questionnaire shortly before (pre) and after (post) the program was delivered. Participating control group (CG) students completed the questionnaire at two time points while attending regular classes, both before receiving the program. Thus, they participated as waitlist-CG.

The pre- and post-test questionnaires were the same for the most part, with the exception that only the pre-test questionnaire requested demographic information and included a scale measuring social desirability. Also, the post-test questionnaire of the IG additionally included items to assess students’ opinions about the program and the degree of acceptability. A detailed description of the students’ reception of the program has already been published [28].

The pre- and post-test administration was partly conducted directly by the project team and partly by instructed teachers when non-school members were not allowed to enter the school due to restrictions related to the COVID-19 pandemic. Prior to study participation, all students were asked to provide written informed consent. Written informed consent of parents or guardians was additionally collected for students younger than 16. The questionnaire did not include personal identifying information at the student or school level. The Ethics Committee of Bielefeld University approved the study.

### Measures

#### Demographic information

Participating students were asked to provide demographic information at pre-test administration, including age, gender, and if they or a person they know (family, friends, acquaintance) experienced a mental illness.

#### Attitudes towards mental illness

Participants’ attitudes towards mental illness were assessed through twelve items. These were adopted from the “Student Survey” questionnaire version of 2017, which was developed by the Canadian creators of *the Guide* (items displayed in Appendix S2 of Wei et al. 2022 [29]). The creators declare that the attitude items in this survey are revised from Youth Opinion Survey. The items were chosen in the present study because they were tailored for the program evaluation. As reported in an evaluation study, the scale’s internal consistency is good (α = 0.82-0.88) [29]. The items were translated into German by the project team. The translation was reviewed via back translation by a native English speaker from the project team and refined according to the feedback. The translation procedure applies to all measures used in this study, as all original items were available in English.

Participating students were asked to rate each item on a 7-point Likert scale indicating how strongly they agree or disagree (answer options: “strongly disagree”, “disagree”, “disagree a little”, “I’m not sure”, “agree a little”, “agree”, “strongly agree”, translating into numerical values of 1 = “strongly agree” to 7 = “strongly disagree”). Total scores were at a minimum of 12 and at a maximum of 84. Higher scores represent more positive attitudes towards mental illness.

The scale comprises two subscales, one consisting of five items addressing “social stigma”, which refers to a person’s misconceptions, negative beliefs, and stigmatizing attitudes about people with mental illness. The second subscale consists of seven items addressing “social distance”, referring to the willingness to interact with people with mental illness [29]. The total scores range for the subscales were 5–35 for social stigma and 7–49 for social distance. In the data analysis, the whole scale and the two subscales will be examined separately to gain more information on the potential effect of the intervention on the different dimensions of stigmatizing attitudes.

#### Self-stigma

A measure of self-stigma was included in the study to assess stigmatizing attitudes more comprehensively, as self-stigma is considered to be an internalized form of public stigmatizing beliefs. Six items measuring participants’ self-stigma were adopted from the “Knowledge and Attitudes to Mental Health (KAMH)” scale, which a Welsh research group developed within a nationwide rollout and evaluation of the Welsh adaption of *the Guide* [30]. The project team contacted the group, and permission to use the items was granted. More information on the KAMH, including psychometric properties, is available, reporting good values on the internal consistency of the self-stigma items (ω = 0.80) [31]. Participants could respond to each of the six self-stigma items on a 5-point Likert scale. The answer options were “strongly agree”, “agree”, “I don’t know”, “disagree”, and “strongly disagree”. The scoring ranges from 0 (= ”strongly disagree”) to 4 (= “strongly agree”), resulting in total sum scores between 0 and 24. In contrast to the scales listed above, higher values, in this case, reflect that more self-stigma is present. See Additional file 1 for the translated (and original) attitude and self-stigma items.

#### Social desirability

As measuring attitudes is amenable to socially desirable responses, the study included a scale to measure and control participants’ degree of social desirability. The eight items used were also adopted from the Welsh KAMH with acceptable internal consistency (α = 0.69) [31]. Participants could respond on a 5-point Likert scale with following the answer options: “strongly agree”, “agree”, “I don’t know”, “disagree”, and “strongly disagree”. The scoring for individual items ranges from 0 to 4, while the total sum score ranges from 0 to 32. Higher scores reflect a higher degree of social desirability.

### Statistical analysis

The students’ responses were eligible for analysis if data were available from both pre- and post-test assessments. Data analysis was done using IBM’s Statistical Package for the Social Sciences (SPSS) version 28 [32]. Descriptive analysis was conducted, including frequencies and mean scores. The sample characteristics of the IG and the CG were tested for differences using independent samples t-test and Chi-squared test.

To determine the most suitable analysis methods for the current data, the intraclass correlation coefficient (ICC) was computed to verify if the data is nested within classes. For none of the scales measuring stigmatizing attitudes, the ICC was significant, ruling out the need for multilevel modeling approaches.

For the attitude (sub-)scales, sum scores were generated if participants responded to all of the items of the respective scale. Pearson correlations were computed to test for potential relationships between attitude outcomes and socially desirable responses for both assessment time points. T-tests for independent samples were conducted to determine sum score differences of attitudes at the pre-test between IG and CG to verify the data comparability.

In order to evaluate the effect of the intervention on the participants’ attitudes towards mental illness before and after the intervention took place, a repeated measures analysis of variance (ANOVA) was performed comparing the pre- and post-test sum scores of the IG and CG. This procedure was applied for each of the (sub-)scales used in this study to measure stigmatizing attitudes. Cohen’s f was calculated to determine the effect sizes of the intervention effect. The f-values can be interpreted as follows: small = > 0.1, medium = > 0.25 and large = > 0.4 [33].

Additionally, the IG’s attitude scores (12-item total scale) were further examined: As the data included was partly not normally distributed, Mann-Whitney-U tests were computed to check for any group differences regarding the IG’s attitude change from pre- to post-test. Group variables used were gender, pre-existing experience with mental illness, and delivery alternative of the contact element (speaker vs. video). The Bonferroni adjusted p-value of statistical significance was α = 0.0167 (α = 0.05/3 = 0.0167).

Regarding scale reliability, internal consistency was measured by Cronbach’s Alpha for each (sub-)scale used in the study.

## Results

### Sample description

The study comprises a convenience sample. Local schools in Bielefeld, Germany, were informed and invited to implement the program in their classes and participate in the evaluation study. Recruitment of participating classes was challenging, mostly due to (i) limited opportunities to implement programs (especially if they require a certain amount of time) in addition to the mandatory curriculum in Germany and (ii) extensive restrictions the COVID-19 pandemic imposed on the schools. In total, seven 10th grade classes from three secondary schools (two schools with intermediate level, one grammar school with higher education level) and two classes from one vocational school participated in the project in the IG between February 2020 and June 2021. Five 10th grade classes of one secondary school (grammar school) participated as CG in the fall of 2021.

The program delivery modalities varied notably due to the limited time resources to implement “add-on” programs and the impact of the COVID-19 pandemic, which led to incomplete program deliveries, month-long interruptions of intervention delivery, and great variance in the time span between pre- and post-test assessment. Given the high variance in the program delivery and corresponding evaluation data, the project team decided to only include the data of one school in this study to reduce confounding factors and ensure a sufficient degree of comparability. Thus, the sample presented in this article consists of five 10th grade classes of one grammar school that participated in the IG in the fall of 2020 and another five 10th grade classes of the same school that took part as CG one year later before receiving the intervention.

The program was delivered within one project day for the included five IG classes. The available documentation of the program delivery shows that in all five classes, the core elements and mandatory activities were completed for the most part, and none of the additional materials were used. All five classes did not complete the last activity of module 6 due to lack of time, and in two classes, one activity was omitted, resulting in an overall high program delivery fidelity rate. Both delivery alternatives of the contact element took place: Two classes were visited by a speaker (direct contact). The remaining three classes used digital stories (videos) included in the program material (indirect contact). Five classroom teachers delivered the program, and two of them attended preparatory training beforehand.

The time period between pre- and post-test administration was about one to two weeks. This was also true for the five classes of the CG who participated in the study one year later.

Data from n = 216 participating students was available from the included ten classes (IG = 5 classes, CG = 5 classes). However, only n = 188 students completed pre- and post-test questionnaires (87.04%). N = 204 only participated in the pre-test, and n = 200 only participated in the post-test. Students who did not complete both pre- and post-test questionnaires were excluded from the sample and further data analysis. Of the included students (n = 188), 106 participated in the IG, whereas 82 students formed the CG.

The mean age of the students included in the study is 15.14 years (SD = 0.560, n = 187). The sample comprises a slightly higher percentage of female participants (58,7%). The demographic characteristics of the IG and CG are similar. There are minor differences in percentages for the gender distribution and the reported experience with mental illness or contact to a person who does. However, conducted χ2-tests show that these differences are not significant. In terms of the mean age, the t-test revealed a significant difference with a medium effect size (d = 0.552; p = < 0.001) between IG and CG. Table 1 shows the sample characteristics.

**Table 1:** Sample characteristics

Characteristics	Total Sample (n = 188)	Intervention group (IG) (n = 106)		Control group (CG) (n = 82)		T-test		
**Age**	*M* = 15.14 (*SD* = 0.560) range 14–17 (n = 187)	*M* = 15.01 (*SD* = 0.561) range 14–17 (n = 106)		*M* = 15.31 (*SD* = 0.516) range 14–17 (n = 81)		t(185)=-3,743, p = < 0.001		
						Chi-squared test		
	*actual*	*actual*	*expected*	*actual*	*expected*	*χ* ^*2*^	*df*	*p*
**Gender**								
Female Male	58.7% (n = 108) 41.3% (n = 76)	57.7% (n = 60) 42.3% (n = 44)	58.7% (n = 61) 41.3% (n = 43)	60.0% (n = 48) 40.0% (n = 32)	41.25% (n = 33) 58.75% (n = 47)	0.099	1	0.753
**Experience with mental illness**								
Yes No/Don’t know/ Don’t want to answer	60.6% (n = 114) 39.4% (n = 74)	63.2% (n = 67) 36.8% (n = 39)	60.7% (n = 64) 39.3% (n = 42)	57.3% (n = 47) 42.7 (n = 35)	60.6% (n = 50) 39.3% (n = 32)	0.627	1	0.412

n = number of subjects, M = mean, SD = standard derivation, *p* = p value, χ2 = Chi² value, df = degrees of freedom

### Outcomes

The Pearson correlations did not show any significant correlation between either pre-test or post-test attitude scores and social desirability for the total sample (IG plus CG) indicating that social desirability is unrelated to the attitude responses (see Additional file 2).

### Attitudes towards mental illness at baseline (pre-test)

The pre-test mean sum scores are 69.24 (SD = 9.41) in the IG and 70.94 (SD = 8.42) in the CG of a maximum score of 84, indicating few stigmatizing attitudes among the participating students at baseline. Regarding the self-stigma items, results show rather neutral levels of self-stigma present for both groups with 12.32 in the IG and 11.11 in the CG (total scores range from 0 to 24, with higher scores representing high levels of self-stigma). Overall, there were no significant differences in the pre-test scores between IG and CG. Thus, the two groups reflect similar baseline scores regarding their degree of stigmatizing attitudes present. The mean baseline sum scores for the scales regarding attitudes and the according test statistics for the differences between IG and CG are shown in Table 2.

**Table 2:** Description of attitude scores at pre-test (i.e. baseline scores)

Sum scores of the pre-test data					T-test
		*n*	*M*	*SD*	
Attitudes towards mental illness (12-item scale)	*IG*	103	69.24	9.41	t(183)= -1.275, p = 0.204
	*CG*	82	70.94	8.42	
Social stigma (5-item subscale)	*IG*	105	27.34	4.04	t(185)= -0.771, p = 0.442
	*CG*	82	27.79	3.85	
Social distance (7-item subscale)	*IG*	104	41.86	6.64	t(184)= -1.359, p = 0.176
	*CG*	82	43.15	6.15	
Self-stigma (6-item scale)	*IG*	106	12.32	4.53	t(185) = 1.714, p = 0.088
	*CG*	81	11.11	5.09	

*IG* = intervention group, *CG* = control group, *n* = number of subjects, *M* = mean, *SD* = standard derivation, *p* = p value

### Intervention effect on attitudes towards mental illness

A repeated measures ANOVA was conducted to compare the attitude scores and their change from pre- to post-test assessment between the IG and the CG to explore the effect of the intervention on attitudes towards mental illness. The results are presented in Table 3. After completing *the Guide* (German version), the sum scores of the 12-item attitudes scale of the participating students in the IG increased significantly (from M = 69.31 to 71.36), whereas a decline was detected in the CG. The ANOVA revealed a statistically significant difference in the attitude change from pre- to post-test between IG and CG (F(1, 181) = 13.744, p = < 0.001) with a medium effect size (f = 0.30). When considering the subscales separately, a significant intervention effect with a medium effect size (f = 0.34) on the social stigma subscale (F(1, 183) = 21.441, p = < 0.001) can be observed. In contrast, no significant change could be noted for social distance. The results of the self-stigma scale show no significant effect of the intervention. Thus, it can be assumed that the contents of the intervention do not influence the degree of self-stigmatizing attitudes.

**Table 3:** Comparison of IG’s and CG’s attitude scores and change from pre- to post-test

	Intervention group (IG)				Control group (CG)							
	Pre-test		Post-test		Pre-test		Post-test		Repeated measures ANOVA			
	*n*	*M (SD)*	*n*	*M (SD)*	*n*	*M (SD)*	*n*	*M (SD)*	*F*	*p*	*Partial η²*	f*
Attitudes towards mental illness (12-item scale)	101	69.31 (9.43)	101	71.36 (10.33)	82	70.94 (8.42)	82	69.48 (9.38)	13.744	**< 0.001**	0.071	0.30
Social stigma (5-item subscale)	103	27.4 (4.04)	103	29.78 (4.35)	82	27.79 (3.85)	82	27.74 (3.90)	21.441	**< 0.001**	0.105	0.34
Social distance (7-item subscale)	104	41.86 (6.64)	104	41.55 (7.26)	82	43.15 (6.15)	82	41.73 (6.71)	2.913	0.090	0.016	
Self-stigma (6-item scale)	106	12.32 (4.53)	106	12.06 (4.86)	81	11.11 (5.09)	81	11.23 (5.08)	0.628	0.429	0.003	

*n* = number of subjects, *M* = mean, *SD* = standard deviation, *F* = F value, *p* = p value (values below 0.05 indicate statistical significance, marked in bold), *η²* = Eta squared, *f* = effect size value according to Cohen (1988); calculated by √(*η²/1-η²)* [33])

*=f was only calculated for significant ANOVA results

The significant positive change in attitudes (12-item attitudes scale) within the IG was further looked at to explore if there are any correlating or influencing variables. Conducted Mann-Whitney U-tests revealed no significant differences regarding the three tested group variables: gender, experience with mental illness, and delivery alternatives of the contact element (speaker vs. video). Numerically, the delivery alternatives showed the greatest difference between the groups: the group that listened to a speaker telling his/her story at class had a bigger positive change (see Additional file 3). Overall, these findings suggest that regardless of students’ gender, experience with mental illness within the social or family context or in oneself, or delivery condition of the contact element, *the Guide* (German version) seemed to have a destigmatizing effect.

### Measurement properties

The internal consistency was measured by Cronbach’s Alpha for every (sub-)scale using the data of the total sample (IG and CG) separately for the pre-test and post-test. Overall, the scales used show mostly acceptable to good values for internal consistency, as depicted in Table 4, with one exception: The value of the subscale social stigma calculated using the pre-test data fell short in reaching an acceptable value. However, the subscale’s value using the post-test data was acceptable.

**Table 4:** Measurement properties

		Pre-test data		Post-test data	
**Measures**	items	*n*	*α*	*n*	*α*
Attitudes towards mental illness	12	185	0.81	186	0.854
Social stigma (subscale)	5	187	0.573	186	0.707
Social distance (subscale)	7	186	0.819	188	0.844
Self-stigma	6	187	0.811	187	0.842
Social desirability	8	183	0.621		

*n* = number of subjects, *α* = Cronbach’s Alpha

## Discussion

### Effect of the Guide (German version) on attitudes towards mental illness

The first-time application of the German version of *the Guide* (an MHL-based intervention) was found partially effective in mitigating stigmatizing attitudes. More precisely, one dimension of public stigma, measured by the social stigma subscale, significantly declined. No significant changes were noted for the social distance subscale and the self-stigma scale. The magnitude of change in the social stigma subscale was so substantial that the total scores reached the significance level when it was considered together with the social distance subscale. Similar results were observed in previous evaluation studies of the application of the Guide in Canada [12–14, 29, 34] and beyond, e.g., Nicaragua [16] or Wales [35], where attitudes towards mental illness were assessed through a composite measure, without looking at the individual contribution of subscales. More precisely, two studies also used a 12-item scale to assess attitudes [29, 34], four studies applied a shorter scale comprising 8 items [12–14, 16], and one used a different set of 6 items to measure stigma (“stigma to others”) [35]. All studies reported positive changes in attitudes comparing pre- and post-test scores with small to medium effect sizes. Some studies included a follow-up assessment that showed the changes would remain stable for two months [12, 13] and one year [29].

Considering the pre-test scores on the subscales social stigma and social distance, it is noteworthy that the attitudes rather fell in the area of non-stigmatizing beliefs, indicating that students did not tend to hold stigmatizing attitudes before the intervention. In contrast to social stigma (IG score at pre-test: 27.4 out of 35), the pre-test scores for social distance were even higher (IG score at pre-test: 41.86 out of 49), leaving less room for substantial improvements in this domain. While the observed ceiling effects seem like a plausible explanation for the absence of a significant positive change for social distance, other possible reasons can be determined by looking at studies that were able to document a positive impact on social distance. Jorm and Oh (2009) corroborated the findings of 16 studies addressing social distance and found that the great majority led to a reduction [25]. However, unlike the MHL intervention used in this study, these interventions focused on specific mental illnesses, such as schizophrenia or depression. Given that the desire for social distance and the degree of stigmatizing attitudes depend on the particular mental illness at hand, the lack of significant change in social distance might also be associated with the more general intervention focus on mental illness. Moreover, the differing effectiveness of interventions on different stigma types was noted before. A meta-analysis of MHL-based interventions found that stigmatizing attitudes improved to a higher degree (d = 0.30) than social distance (d = 0.16) [9].

With regards to self-stigma, the analysis did not show a significant decrease following the delivery of *the Guide* (German version). Generally, little is known about the effects of universal or general mental health/MHL/stigma interventions on self-stigma, as it is often not measured [36]. Mostly, this is also the case for the *the Guide* intervention. Until now, only the study that evaluated the application of the Welsh version of *the Guide* measured self-stigma [31, 35]. The Welsh study did find an improvement in self-stigma in the IG. However, the scores of the CG improved as well. Thus, it is unclear whether the intervention produced the improvements. Also, the pre-test self-stigma scores in the present study and those in the Welsh study are comparable, as both measured scores fall in the neutral realm.

As self-stigma is described to be the internalization of public stigma, the development of measurable self-stigma requires (i) being aware of public stigma towards people with mental illness, (ii) agreeing with the negative beliefs or stereotypes, and (iii) applying these to oneself, meaning a self-identification with the stereotyped group [37]. The neutral self-stigma scores in the study might be attributed to the lack of identification with the group of “people with mental illness”. Given the universal nature of the intervention, it is likely that only a few participants experienced mental health issues explaining the low levels of self-stigma at pre-test. Future studies could collect data about the participants’ mental health status and compare how self-stigma changes for adolescents with pre-existing mental health problems and those without.

The intervention’s potential for destigmatization can also be derived from the findings of the students’ subjective assessment of *the Guide* (see [28]). When asked why participants thought the program was helpful for students their age, they responded that it addressed an unmet need for more awareness regarding stigma and how to tackle it [28].

### Potential associates of the change in attitudes towards mental illness

The improvements in attitudes in the IG were not related to differences in gender, pre-existing experience with mental illness, and the delivery modality of the contact element. Although the mean scores varied, e.g., females, students with pre-existing experience, and those who had listened to a speaker (direct contact), have had higher scores at pre- and post-test assessment and a slightly higher degree of change from pre- to post-test, the changes did not differ significantly (see Additional file 3). This indicates that the program positively affected the attitudes of male and female students, students with and without pre-existing experience, and those who experienced direct and indirect contact within the intervention alike.

Regarding gender differences, a recent Canadian evaluation study on *the Guide* also showed that immediately after the intervention, there were no significant differences between students who identified as male compared to female, however, the change in attitudes at one-year follow-up was significantly greater for females compared to male students [29].

With respect to pre-existing experience with mental illness, it can be presumed, that being more knowledgeable about mental illness or knowing someone with a mental health problem might be associated with greater intervention effects, because these people might “already agree with the message” (p.353) [38]. Even though the stigma change of the group with pre-existing experience was greater in this study, the results fell short of reaching statistical significance. Thus, the study cannot support the assumption that pre-existing experience with mental illness contributes to greater stigma reduction.

To our knowledge, the study is the first to compare contact delivery alternatives when applying the *the Guide* intervention. Including a contact session with a person with lived experience of mental illness is generally thought to be an important and largely used element of interventions targeting stigma. However, the effectiveness of varying delivery methods of contact (direct or indirect) has not been conclusively determined yet. Both indirect and direct contact seem to positively influence stigmatizing attitudes, with direct, in-person contact being argued to be more effective [39, 40]. The results of this study are predominantly consistent with prior research, as both alternatives yield positive effects. The results do not confirm that in-person contact has a significantly greater effect. Based on the findings, both alternatives of contact delivery (video-based vs. in-person) can be recommended when applying *the Guide* (German version), however, this finding should be viewed within the scope of this exploratory study. When considering the involvement of speakers with lived experience with mental illness (direct contact), schools should be aware of the according organizational efforts (e.g., acquisition of speakers, scheduling, preparation, etc.).

The results must be considered cautiously as the sample size might not be sufficient for group comparison. More extensive research is necessary to confirm the findings and also to explore further (i) gender differences of the short- and long-term effects of *the Guide* and its German version on attitudes, (ii) how pre-existing experience might factor in how participants respond to and benefit from the intervention and (iii) the modality of how contact is being delivered (indirect, video-based contact vs. direct, in-person contact) in comparison studies, to gain evidence regarding which alternative makes the *the Guide* intervention most successful in reducing stigma.

### Strengths and limitations

Methodological strengths of the study are, for one, the inclusion of a control group, in which no comparable effect was measured. Therefore, the effect found in the IG can strongly be tied to program participation. Additionally, social desirability was controlled for, showing that none of the attitude scores significantly correlated with the social desirability scores, which also favors the validity of the findings. Another benefit is the use of measures that are tailored to *the Guide* and have been applied before to evaluate the effectiveness of *the Guide* in different countries, which enables cross-country comparisons of results. As reported above, the internal consistency of the translated (sub-)scales was mostly acceptable and good, which speaks for the validity of the translated items.

Moreover, this study intended to look into the intervention effect of *the Guide* (German version) on stigmatizing attitudes more comprehensively. Besides including a measure for self-stigma, the scale to measure attitudes towards mental illness (12-item scale) which assesses two different domains of public stigma, social stigma (personal stigmatizing beliefs about people with mental illness, items 1–5) and social distance (stigmatizing behavioral intentions, items 6–12), was analyzed in two ways: first the overall scores were calculated and analyzed just like other studies on *the Guide* did in the past. Secondly, the items representing each domain were aggregated into two subscales and analyzed separately, which has not been done previously. This method allows for more information as to whether the destigmatizing effect of *the Guide* (German version) is different for varying stigma domains.

The use of a convenience sample and the relatively small sample size limit the generalizability of the results. Moreover, the data included in the study is rather homogenous, as participating students originated from one school and one age group (10th grade) only. Although this increases the comparability of the IG and CG, it limits the transferability of the findings to other school types and age groups. Thus, whether the intervention has similar effects among younger students (e.g., grade 8 or 9) or students visiting other school types (secondary schools in Germany are divided by academic level and practical orientation) remains unclear.

Because of the limited capacities of schools to organize and conduct extra-curricular programs and participate in evaluation studies during the COVID-19 pandemic, the initially planned follow-up assessment could not be realized. Thus, there is no evidence if the measured attitude changes would sustain over time. Due to sample size and data quality, multivariate analysis was not feasible as the data did not meet the necessary criteria.

### Future research directions

This exploratory study offers first insights and starting points for future research on *the Guide* (German version). Due to the exploratory nature of the study, the particular circumstances the intervention took place in (COVID-19 pandemic), and the resulting small, rather specific sample, as mentioned before, there is a need for replication studies to confirm the findings and augment the evidence base. In particular, it would be important to expand the variety of target groups in the sample and look into, e.g., different age groups, minorities, and high-risk groups, to document the potential of the intervention in a more differentiated way. Thus, more extensive studies with representative samples, and robust study designs (RCT ideally), including follow-up assessments, would be important to verify the findings and allow multivariate analysis to explore further and determine factors that influence the program’s effectiveness with regards to decreasing different forms of stigma as well as potential catalysts that support stigma reduction.

Moreover, since the stigma of mental illness is a societal problem, raising awareness among specific groups like students, or professionals is not enough. Other approaches should address the general public. In addition, the effects of structural stigma and its interaction with individual forms of stigma need to be acknowledged and addressed. The availability of the *the Guide* intervention in different languages and countries would allow for a cross-cultural analysis where structural aspects of stigma could be considered as well. Future studies should investigate how much change can an individual-focused intervention yield, depending on how discriminatory laws and legislations in a particular region are towards people with mental illness or on how people with mental illness are portrayed as in the media (i.e., are they depicted as “violent” and “unpredictable” or is their issue normalized and put into perspective). While decreasing stigmatizing attitudes of the population is a relevant area of mental health action, efforts to address structural stigma need to concomitantly happen to evoke meaningful shifts in public attitudes.

## Conclusions

The study shows that the MHL-based intervention *the Guide* could be adapted for application in Germany and documents the intervention’s promising effect regarding decreasing stigmatizing attitudes, in particular regarding social stigma, among German 10th grade students in grammar school. The intervention’s potential needs to be further examined.

### Electronic supplementary material

Below is the link to the electronic supplementary material.

Supplementary Material 1

Supplementary Material 2

Supplementary Material 3

## Data Availability

The dataset generated and analyzed during the current study is not publicly available as participants’ informed consent did not include sharing the raw data publicly, but is available from the corresponding author on reasonable request.

## Footnotes

1 Various versions of *the Guide*, including the original and the German version, can be found here: https://mentalhealthliteracy.org/product/mental-health-high-school-curriculum/. *The Guide* (German version) plus teaching materials can additionally be accessed here: https://www.uni-bielefeld.de/fakultaeten/erziehungswissenschaft/zpi/projekte/downloads/.

## Abbreviations

ANOVA: Analysis of variance
CG: Control Group
ICC: Intraclass correlation coefficient
IG: Intervention group
KAMH: Knowledge and attitudes to mental health
MHL: Mental health literacy
SPSS: Statistical package for the social sciences
