# Supplementary material for: Evaluating the effect of an adapted mental health literacy intervention on mental health related stigma among secondary students in Germany: results of a pre-post evaluation study
Source: BMC Public Health. 2023 Oct 10;23:1959. doi: 10.1186/s12889-023-16825-y (PMC10563208; doi:10.1186/s12889-023-16825-y)
Supplement: Supplementary file 1 — Supplementary Material 1 [file 12889_2023_16825_MOESM1_ESM.docx]

Additional file 1

Additional file 1 comprises two tables displaying the items used in the study to access stigmatizing attitudes of the participating students.

Table 1: Attitude items (adopted from “Student Survey” Questionnaire (Version 2017))^1^

|  | **Original items (English)** | **Translated items (German)** |
| --- | --- | --- |
| 1 | Most people with a mental illness are too disabled to work. | Die meisten Menschen mit einer psychischen Erkrankung sind zu beeinträchtigt, um zu arbeiten. |
| 2 | People with a mental illness tend to bring it on themselves. | Menschen mit einer psychischen Erkrankung sind gewöhnlich selbst schuld daran. |
| 3 | People with mental illnesses don’t try hard enough to get better. | Menschen mit einer psychischen Erkrankung strengen sich nicht genug an, damit es ihnen besser geht. |
| 4 | Most violent crimes are committed by people with a mental illness. | Die meisten Gewalttaten werden von Menschen mit einer psychischen Erkrankung begangen. |
| 5 | You can’t rely on people with a mental illness. | Man kann sich nicht auf Menschen mit einer psychischen Erkrankung verlassen. |
| 6 | I would be upset if someone with a mental illness always sat next to me in class. | Es würde mich ärgern, wenn in der Klasse jemand mit einer psychischen Erkrankung immer neben mir sitzen würde. |
| 7 | I would not be close friends with someone I knew with a mental illness. | Ich würde nicht mit jemandem, den ich kenne, eng befreundet sein, wenn sie/er eine psychische Erkrankung hat. |
| 8 | If I knew someone had a mental illness, I would not date them. | Wenn ich wüsste, dass jemand eine psychische Erkrankung hat, würde ich nicht mit ihr/ihm auf ein Date gehen. |
| 9 | I would not want to be taught by a teacher who had been treated for a mental illness. | Ich würde nicht von einer Lehrerin/einem Lehrer unterrichtet werden wollen, die/der wegen einer psychischen Erkrankung behandelt wurde. |
| 10 | I would tutor a classmate who got behind in their studies because of a mental illness.* | Ich würde einer Mitschülerin/einem Mitschüler, die/der mit dem Lernstoff wegen einer psychischen Erkrankung hinterher hängt, Nachhilfe geben. |
| 11 | I would not mind it if someone with a mental illness lived next door to me.* | Ich hätte nichts dagegen, wenn jemand mit einer psychischen Erkrankung direkt nebenan wohnt. |
| 12 | I would avoid someone with a mental illness. | Ich würde jemanden mit einer psychischen Erkrankung aus dem Weg gehen. |
| ^1^Wei Y, Church J, Kutcher S. Long-term impact of a mental health literacy resource applied by regular classroom teachers in a Canadian school cohort. Child Adolesc Ment Health 2022. doi:10.1111/camh.12597 (Appendix S2)  Items 1-5 form the subscale “social stigma”, items 6-12 form the subscale “social distance”  * Item was reverse coded before entering data analysis | | |

Table 2: Self-stigma items (adopted from the KAMH²)

|  | **Original items (English)** | **Translated items (German)** |
| --- | --- | --- |
| 1 | If I had a mental disorder, I would feel worthless like I had failed my family. | Wenn ich eine psychische Erkrankung hätte, würde ich mich wertlos fühlen, so als wenn ich meine Familie enttäuscht hätte. |
| 2 | If I had a mental disorder I would not avoid socialising.* | Wenn ich eine psychische Erkrankung hätte, würde ich nicht vermeiden, unter Menschen zu gehen. |
| 3 | I would feel a failure if I had a mental disorder. | Ich würde mich wie eine Versagerin/ein Versager fühlen, wenn ich eine psychische Erkrankung hätte. |
| 4 | If I had a mental disorder I would not feel ashamed.* | Wenn ich eine psychische Erkrankung hätte, würde ich mich nicht schämen. |
| 5 | I would feel weak if I had a mental disorder. | Ich würde mich schwach fühlen, wenn ich eine psychische Erkrankung hätte. |
| 6 | If I had a mental disorder, I would feel I’d let everyone down. | Wenn ich eine psychische Erkrankung hätte, hätte ich das Gefühl, alle zu enttäuschen. |
|  | ²Simkiss, N.J.; Gray, N.S.; Dunne, C.; Snowden, R.J. Development and psychometric properties of the Knowledge and Attitudes to Mental Health Scales (KAMHS): a psychometric measure of mental health literacy in children and adolescents. BMC Pediatr. 2021, 21, 508, doi:10.1186/s12887-021-02964-x  * Item was reverse coded before entering data analysis | |
