# Supplementary material for: Evaluating the effect of an adapted mental health literacy intervention on mental health related stigma among secondary students in Germany: results of a pre-post evaluation study
Source: BMC Public Health. 2023 Oct 10;23:1959. doi: 10.1186/s12889-023-16825-y (PMC10563208; doi:10.1186/s12889-023-16825-y)
Supplement: Supplementary file 2 — Supplementary Material 2 [file 12889_2023_16825_MOESM2_ESM.docx]

**Additional file 2**

Additional file 2 comprises a table displaying the results of Pearson correlation calculated for attitude and social desirability scores.

Table: Pearson correlation calculated for attitude and social desirability scores (total sample, IG+CG)

| **Social desirability^1^** | Pretest | | | Posttest | | |  |
| --- | --- | --- | --- | --- | --- | --- | --- |
|  | *n* | *r* | *p* | *n* | *r* | *p* |  |
| Attitudes towards mental illness (12 items) | 181 | .062 | .405 | 181 | .084 | .260 |  |
| Social stigma (subscale, items 1-5) | 183 | .009 | .900 | 181 | .037 | .619 |  |
| Social distance (subscale, items 6-12) | 181 | .082 | .273 | 183 | .100 | .179 |  |
| Self-stigma (6 items) | 183 | -.118 | .113 | 183 | -.095 | .200 |  |
| *^1^* Social desirability was only measured at pre-test. Bivariate correlations were computed between pre-test social desirability scores and pre- as well as post-test scores of all attitude (sub-)scales.  *n*=number of subjects, *r*=Pearson correlation coefficient, *p*=p-value | | | | | | | |
