# Supplementary material for: Evaluating the effect of an adapted mental health literacy intervention on mental health related stigma among secondary students in Germany: results of a pre-post evaluation study
Source: BMC Public Health. 2023 Oct 10;23:1959. doi: 10.1186/s12889-023-16825-y (PMC10563208; doi:10.1186/s12889-023-16825-y)
Supplement: Supplementary file 3 — Supplementary Material 3 [file 12889_2023_16825_MOESM3_ESM.docx]

**Additional file 3**

Additional file 3 comprises a table of the Mann-Whitney U-test results comparing the intervention group’s attitude change alongside the group variables gender, experience with mental illness, and delivery alternative of contact.

Table: IG’s attitude scores divided by group variables (gender, experience with mental illness, delivery alternative of contact), and attitude change tested for significant differences by Mann-Whitney U-test

| **Attitude scores of IG (12-item attitudes towards mental illness scale)** | | | | | |
| --- | --- | --- | --- | --- | --- |
|  | Pre-test | Post-test | Change in attitudes  (difference: posttest-pretest) | Mann-Whitney U | |
| **Gender** |  |  |  | *U* | *p* |
| Female (n=56) | M=72.54 (SD=8.19) | M=74.71 (SD=7.57) | 2.18 (SD=4.89) | 1154.5 | .726 |
| Male (n=43) | M=64.93 (SD=9.45) | M=66.70 (SD=11.81) | 1.77 (SD=9.41) |  |  |
| **Experience with mental illness** |  |  |  |  |  |
| Yes (n=63) | M=71.94 (SD=8.36) | M=74.11 (SD=7.91) | 2,17 (SD=5.01) | 1153.5 | .760 |
| No/Don’t know/ Don’t want to answer (n=38) | M=64.95 (SD=9.59) | M=66.79 (SD=12.21) | 1.84 (SD=9.72) |  |  |
| **Delivery alternative of contact** |  |  |  |  |  |
| Speaker (direct contact) (n=37) | M=70.76 (SD=9.27) | M=72.73 (SD=8.44) | 1.97 (SD=7.21) | 893.0 | .391 |
| Digital story (indirect contact) (n=54) | M=68.07 (SD=9.65) | M=69.30 (SD=11.73) | 1.22 (SD=6.73) |  |  |
| IG=intervention group, n=number of subjects, M=mean, SD=standard derivation, *U*=Mann-Whitney U-test value, *p*=p-value | | | | | |
